# Supplementary material for: Ex Vivo Analysis of Cell Differentiation, Oxidative Stress, Inflammation, and DNA Damage on Cutaneous Field Cancerization
Source: Int J Mol Sci. 2024 May 26;25(11):5775. doi: 10.3390/ijms25115775 (PMC11171589; doi:10.3390/ijms25115775)
Supplement: Supplementary file 1 [file ijms-25-05775-s001.zip › ijms-2986682-supplementary/Supplementary materials.pdf]

Supplementary materials

Supplementary Table S1. Antibodies used for western blotting and IHC.

**Antibody**	**Host animal**	**Diluition for western blot**	**Diluition for IHC/IF**	**Distributor**	**Cat. num**
Anti-8OHdG	Rabbit	/	1:300	Bioss Antibodies, Woburn, MA, USA	bs-1278R
Anti-CK10	Mouse	1:1000	1:150	Thermo Fisher, Waltham, MA, USA	MA1-5766 (AE20 clone)
Anti-CK14	Mouse	1:1500	1:200	Abcam, Cambridge, UK	ab7800 (LL02 clone)
Anti-Filaggrin	Mouse	1:500	1:50	Abcam, Cambridge, UK	ab17808 (SPM181 clone)
Anti-iNOS	Rabbit	1:2000	1:200	Thermo Fisher, Waltham, MA, USA	PA3030A
Anti-Ki67	Mouse	/	1:50	Abcam, Cambridge, UK	ab8191 (B126.1 clone)
Anti-OGG1	Rabbit	1:1000	1:100	Thermo Fisher, Waltham, MA, USA	PA5-98026
Anti-p16	Mouse	/	1:50	Merck-Millipore, Burlington, MA, USA	MABE1328 (13H4.1 clone)
Anti-p21	Rabbit	/	1:50	Merck KGaA, Darmstadt, Germany	SAB1306168
Anti-p53	Rabbit	1:500	1:200	Merck-Millipore, Burlington, MA, USA	SAB4504499
Anti-SOD-1	Mouse	1:200	1:100	Santa Cruz Biotechnology, Dallas, TX, USA	sc-374205 (D7 clone)
Anti-β-actin	Mouse	1:5000	/	Thermo Fisher, Waltham, MA, USA	MA1-140 (15G5A11/E2 clone)

Supplementary Table S2. Primer sequences

Gene name	Forward primer (5'-3')	Reverse primer (5'-3')
CK-10	GCATGGCAACTCACATCAGG	CAGCCTGGCATTGTCGATCT
CK-14	CATGAGTGTGGAAGCCGACAT	GCCTCTCAGGGCATTCATCTC
Filaggrin	CAGGATGAAGCCTATGACACCA	TGCAATGGTACCTGGCTTGT
GAPDH	AACGTGTCAGTGGTGGACCTG	AGTGGGTGTCGCTGTTGAAGT
IL-10	CATCGATTTCTTCCCTGTGAA	TCTTGGAGCTTATTAAAGGCATTC
IL-1β	ACAGATGAAGTGCTCCTTCCA	GTCGGAGATTCGTAGCTGGAT
IL-6	GGAGACTTGCCTGGTGAAAA	GTCAGGGGTGGTTATTGCAT
IL-8	ATGACTTCCAAGCTGGCCGT	TCCTTGGCAAAACTGCACCT
iNOS	GTTCTCAAGGCACAGGTCTC	GCAGGTCACTTATGTCACTTATC
Ki-67	CCAGCACGTCGTGTCTCAA	TGAGTCATCTGCGGTACTGTC
OGG1	GCGACAAGACCCCATCGAAT	CCGGAAAAAGTTTCCAGCCAG
p16	CATAGATGCCGCGGAAGGT	AAGTTTCCCGAGGTTTCTCAGA
p21	TGGAGACTCTCAGGGTCGAAA	GGCGTTTGGAGTGGTAGAAATC
SOD-1	GTGGGCCAAAGGATGAAGAGA	ATAGACACATCGGCCACACC
TP53	ACCTATGGAACTACTTCCTGAAA	GAGCTTCATCTGGACCTGGG
