# Supplementary material for: Ex Vivo Analysis of Cell Differentiation, Oxidative Stress, Inflammation, and DNA Damage on Cutaneous Field Cancerization
Source: Int J Mol Sci. 2024 May 26;25(11):5775. doi: 10.3390/ijms25115775 (PMC11171589; doi:10.3390/ijms25115775)

Figure 1d: anti-p53

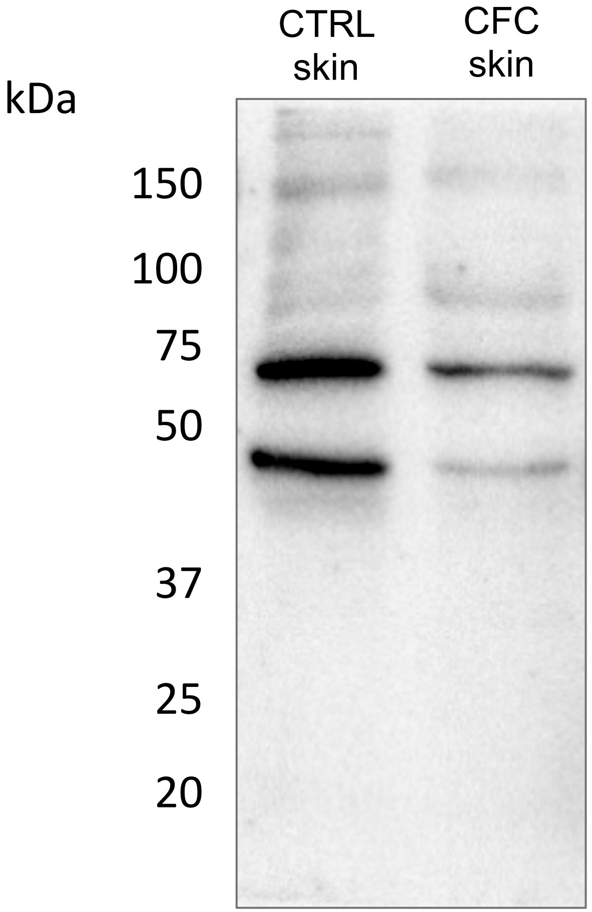

Figure 1d: anti-β-actin

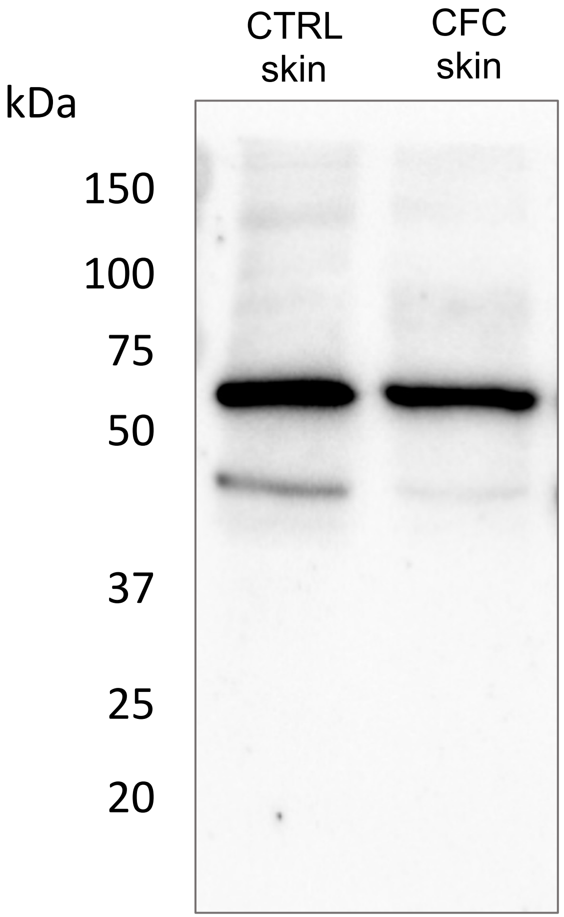

Figure 3e: anti-CK14

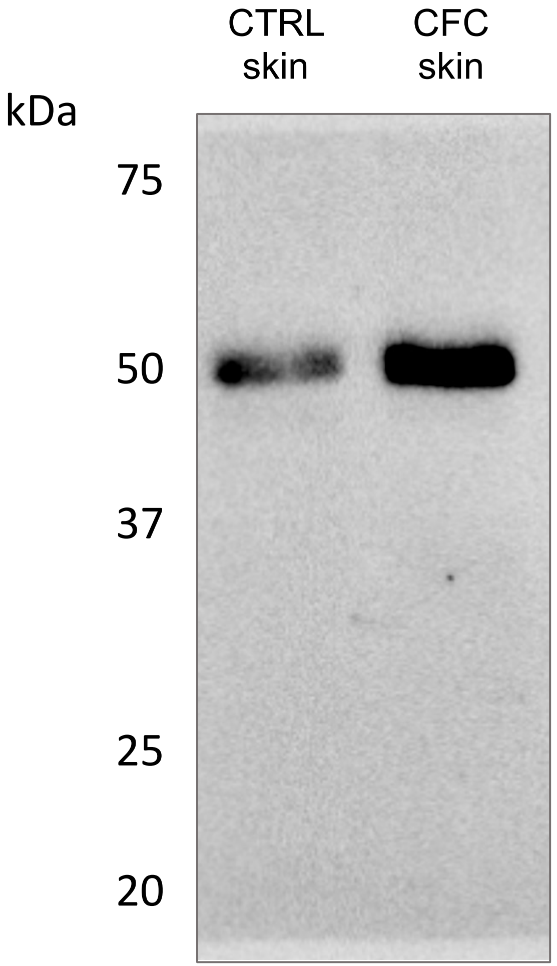

Figure 3e: anti-β-actin

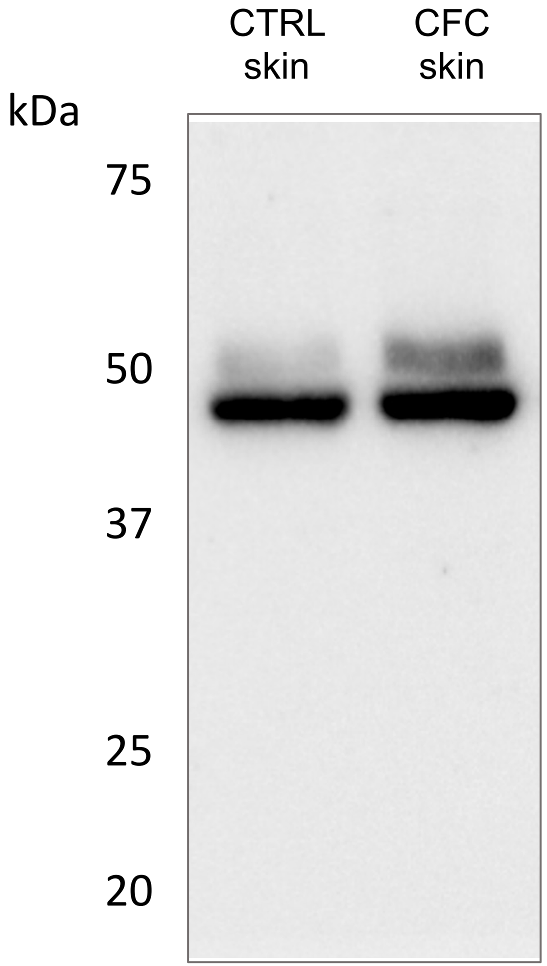

Figure 3e: anti-CK10

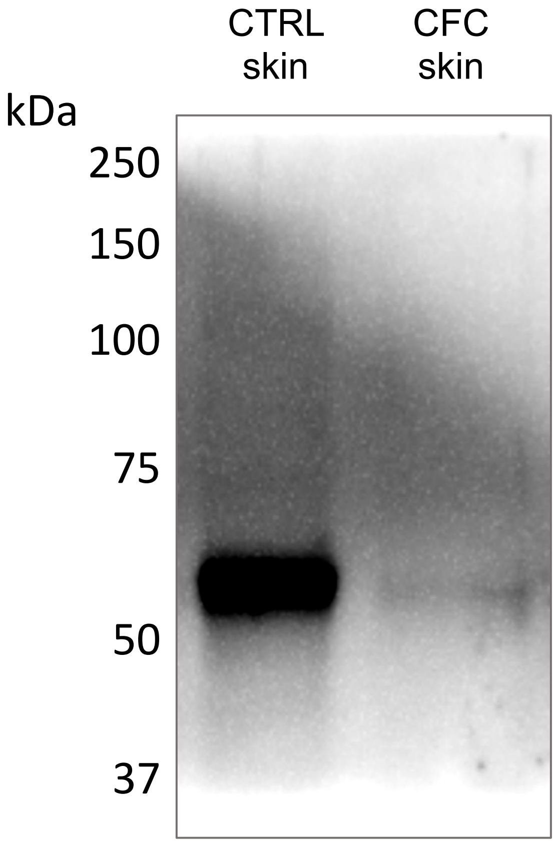

Figure 3e: anti-β-actin

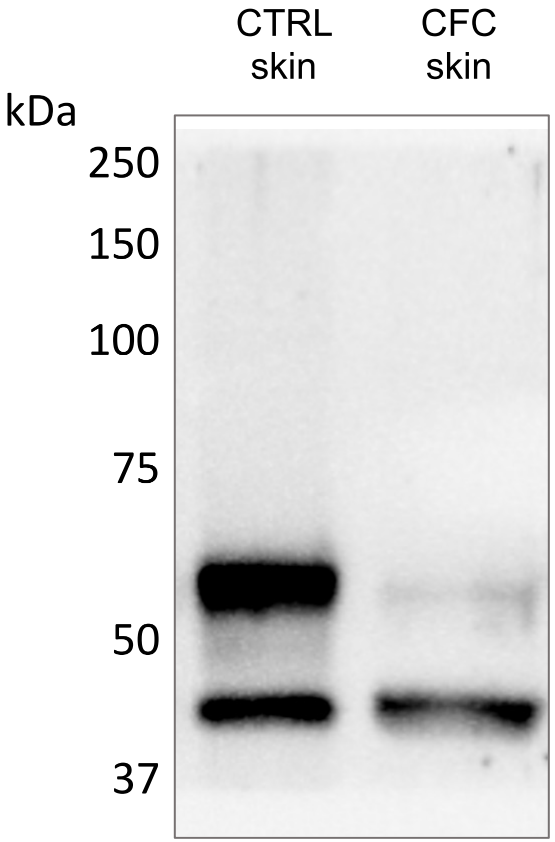

Figure 3e: anti-Filaggrin

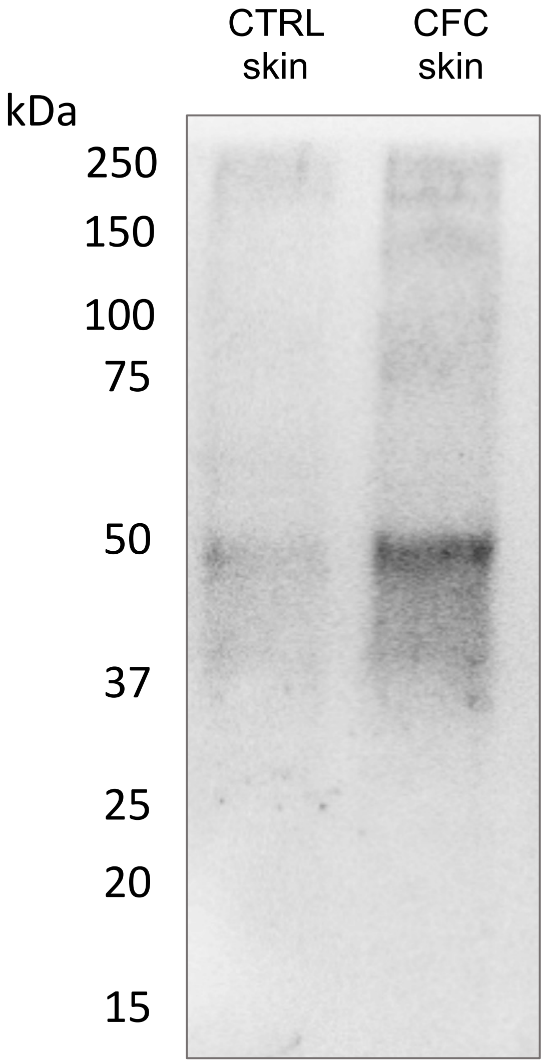

Figure 3e: anti-β-actin

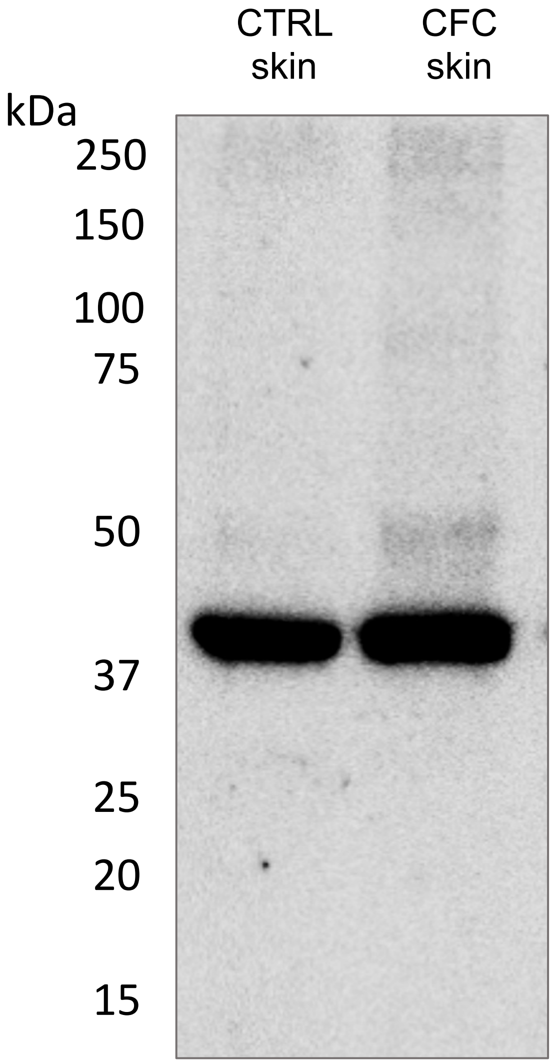

Figure 4e: anti-SOD1

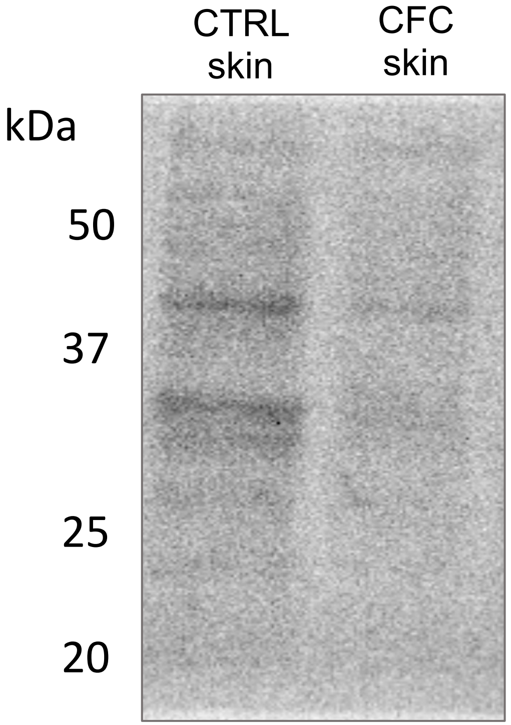

Figure 4e: anti-iNOS

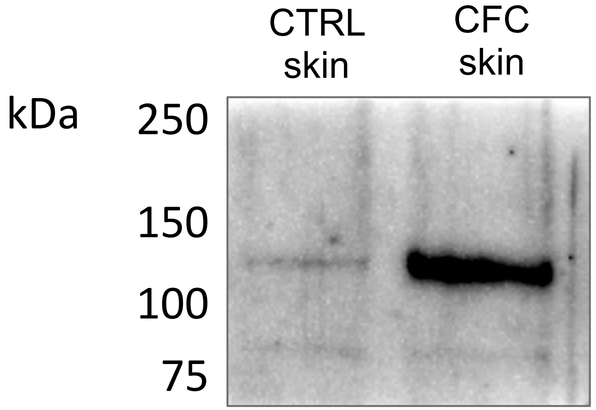

Figure 4e: anti-β-actin

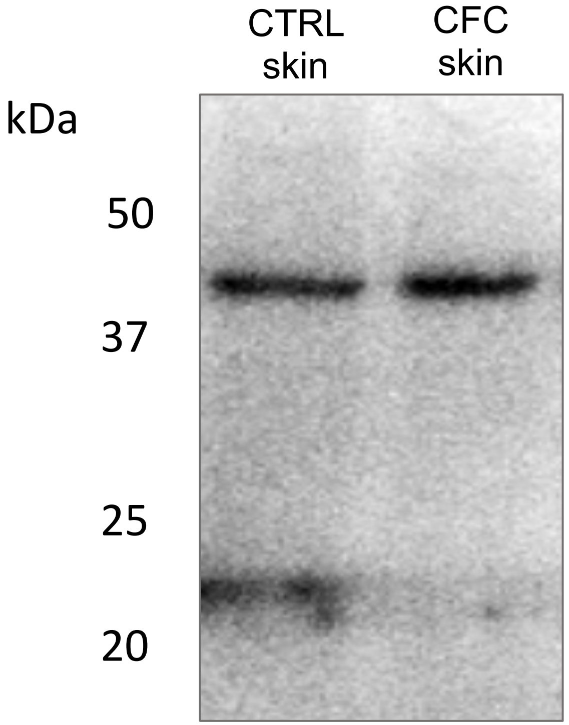

Figure 5f: anti-OGG1

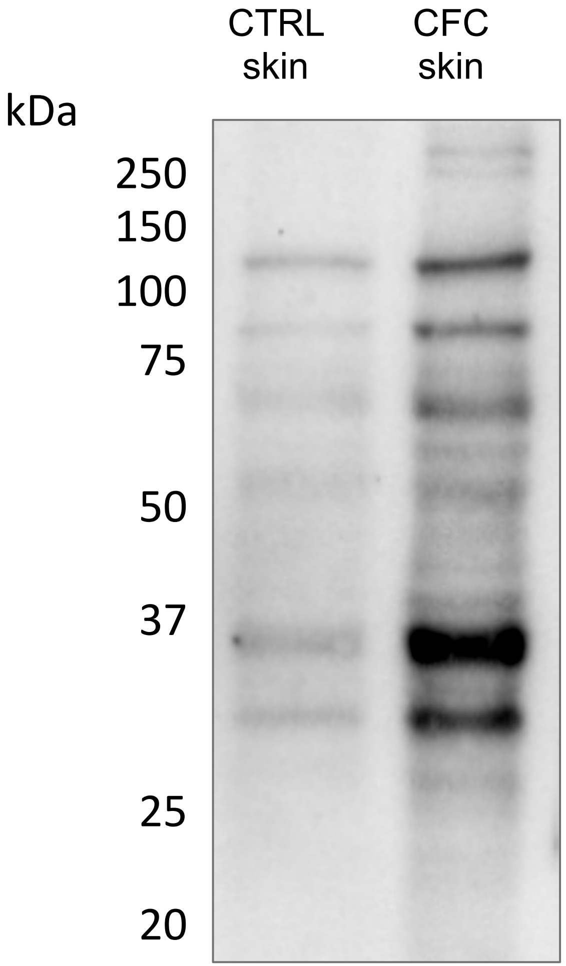

Figure 5f: anti-β-actin

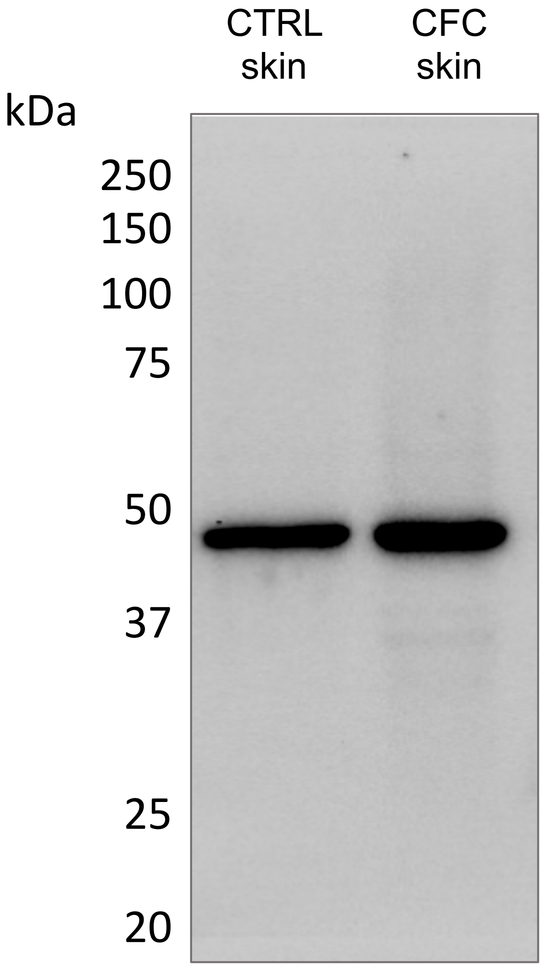

Supplement: Supplementary file 1 [file ijms-25-05775-s001.zip › ijms-2986682-supplementary/Supplementary S1_western blotting.pdf]
